# Wild *Cordyceps sinensis* exhibits far lower arsenic accumulation and hepatorenal toxicity in mice compared to equivalent dose of inorganic arsenic

**DOI:** 10.3389/fphar.2025.1625045

**Published:** 2025-06-24

**Authors:** Liang Gao, Hongxia Yang, Jinmei Ma, Hongtao Bi, Yuancan Xiao, Cen Li, Lixin Wei

**Affiliations:** 1 Qinghai Provincial Key Laboratory of Tibetan Medicine Pharmacology and Safety Evaluation, Northwest Institute of Plateau Biology, Chinese Academy of Science, Xining, China; 2 CAS Key Laboratory of Tibetan Medicine Research, Northwest Institute of Plateau Biology, Chinese Academy of Sciences, Xining, China; 3 University of Chinese Academy of Sciences, Beijing, China; 4 Department of Pathobiology, School of Veterinary Medicine, University of Pennsylvania, Philadelphia, PA, United States

**Keywords:** wild Cordyceps sinensis, inorganic arsenic, safety, arsenic accumulation, liver and kidney damage

## Abstract

**Introduction:**

Wild Cordyceps sinensis (*C. sinensis*) is a Chinese medicinal material known for its renal and pulmonary benefits. However, inorganic arsenic in wild Cordyceps sinensis may accumulate in the body following prolonged consumption; therefore, rigorous safety evaluations are needed.

**Methods:**

This study compared the impacts of wild Cordyceps sinensis at the maximum clinical dose and equivalent doses of inorganic arsenic (16.36 mg/kg) to its total arsenic dose on organ indices, arsenic accumulation, and functional and pathological changes in the liver and kidney in mice, aiming to explore the safety of consuming wild Cordyceps sinensis. Arsenic accumulation in organs was measured via ICP–MS, while serum markers of liver and kidney functions were assessed via ELISA and biochemical assay kits. Histopathology was observed through H&E staining.

**Result:**

Compared with those in the control group, no significant adverse effects on body weight, organ indices, arsenic accumulation, liver or kidney function, or liver or kidney pathology were observed in the Cordyceps group. In contrast, inorganic arsenic exposure resulted in significant arsenic accumulation in organs, especially in the liver and kidneys (*p* < 0.01), liver and kidney function impairment (*p* < 0.01), and pathological changes, including hepatic steatosis, mild edema, balloon degeneration, and renal tubular epithelial cell edema and degeneration, with the aggregation of eosinophils in the renal capsule.

**Conclusion:**

These findings indicate that, at the maximum clinical dose, wild Cordyceps sinensis does not cause measurable hepatorenal toxicity in long-term and exhibits markedly greater safety compared to a mixture of inorganic arsenic compounds (sodium arsenate and sodium arsenite) at an equivalent total arsenic dose.

## 1 Introduction

Wild *C. sinensis* (*Cordyceps sinensis*) is a medicinal fungus belonging to the Clavicpitaceae family that parasitizes the larvae of *Lepidopteran* insects (Committee, 2020). It is distributed primarily in the high-altitude grassland regions of the Tibetan Plateau (Yi et al., 2011). Traditionally, wild *C. sinensis* has been recorded for tonifying the kidneys and lungs and for preventing bleeding and resolving phlegm (Committee, 2020). Studies indicate that it possesses immune-modulatory, anticancer, lung and liver-kidney protective, antioxidant, and antiaging effects (Chen et al., 2013; Fu et al., 2019; Ong and Aziz, 2017; Zhang et al., 2021). However, the detection of relatively high arsenic levels (2.10–9.97 mg/kg) in wild *C. sinensis* (Wang et al., 2008) has raised substantial safety concerns regarding its chronic consumption.

The National Medical Products Administration of China reported that the total arsenic content in wild *C. sinensis* ranged from 4.4 to 9.9 mg/kg, and long-term consumption may lead to arsenic accumulation in the body, thus increasing health risks (NMPA, 2016). Xiao et al. further detected arsenic levels of 5.77–13.20 mg/kg in wild *C. sinensis*, reinforcing concerns about arsenic accumulation with chronic intake (Xiao et al., 2021). Chronic arsenic exposure at low doses typically results in progressive bioaccumulation in various organs, particularly causing severe damage to the liver and kidneys. Mechanistically, arsenic accumulation induces oxidative stress, cellular apoptosis, and steatosis in hepatic cells, ultimately resulting in liver dysfunction, cirrhosis, or hepatocellular carcinoma. Renal arsenic accumulation often leads to renal tubular injury, impaired renal filtration, and even chronic renal failure and fibrosis (Abdul et al., 2015; Centeno et al., 2006; Chen et al., 2024; Santra, 2015; Zheng et al., 2014). The toxicity of arsenic is closely related to its chemical form. Organic arsenicals exhibit low toxicity and negligible bioaccumulation. Among inorganic arsenic species, trivalent arsenic (AsIII) has the highest toxicity; however, although it is less toxic, pentavalent arsenic (AsⅤ) still poses significant health risks (Hughes, 2002). Studies have shown that the arsenic in wild *C. sinensis* is primarily inorganic arsenic, which is more toxic, accounting for 8.69% of the total arsenic, underscoring potential toxicity concerns (Zhou et al., 2018; Zuo et al., 2018).

Given these risks, regulatory authorities worldwide have implemented strict arsenic limits in pharmaceuticals and foods. The Chinese Pharmacopoeia (Committee, 2020) allows a maximum total arsenic content of 2.0 mg/kg in herbal medicines, whereas the European Pharmacopoeia (HealthCare, 2017) sets the limit at 1.5 mg/kg for pharmaceutical products. The International Council for Harmonization of Technical Requirements for Medicinal Products for Human Use (ICH) has established a limit of 0.015 mg/day for oral exposure to inorganic arsenic (ICH, 2022). In addition, the World Health Organization (WHO) recommends a maximum arsenic concentration of 0.01 mg/L in drinking water (WHO, 2011), whereas the European Union and China’s National Food Safety Standards set the limit for inorganic arsenic in rice at 0.20 mg/kg (Commission, 2006; Wu, 2012). The arsenic levels of wild *C. sinensis* exceed these regulatory thresholds, raising concerns about the potential health risks of long-term consumption. Despite these concerns, most studies have focused on determining the total arsenic content and speciation in wild *C. sinensis*, often using the U.S. EPA’s long-term exposure carcinogenic and noncarcinogenic risk models (Xiao et al., 2021). To date, no study has provided direct experimental evidence of *in vivo* organ accumulation from wild *C. sinensis* or the resulting hepatotoxic and nephrotoxic effects.

This study addresses the lack of direct experimental evidence on arsenic accumulation and related toxicity in wild *C. sinensis*, we carried out an *in vivo* study in CD-1 mice. They were orally administered either the maximum clinical dose of wild *C. sinensis* (representing the highest arsenic exposure attainable in a single dose) and an equivalent dose of inorganic arsenic compounds, representing the worst-case toxicological scenario. We analyses of organ-specific arsenic accumulation, organ indices, serum liver/kidney function biomarkers, and hepatic/renal histopathology. Collectively, these comparative data demonstrate that *wild C. sinensis* causes no detectable hepatorenal toxicity in mice at the maximum clinical dose in long-term and shows substantially safer than an equivalent total arsenic dose of inorganic arsenic compounds mixture (sodium arsenate and sodium arsenite), providing reference evidence for understanding its dietary safety.

## 2 Materials and methods

### 2.1 Materials and reagents

Wild *C. sinensis* (*C. sinensis*) was harvested from Zaduo County, Yushu Prefecture, Qinghai Province. Sodium arsenite (840622, AR, purity: 95%) and trisodium arsenate (880714, AR, purity >98%) were purchased from Beijing 5,761 Chemical Plant and Shanghai Chemical Reagent Procurement and Supply Station. The enzyme-linked immunosorbent assay (ELISA) kits for AST (JL13992) and ALT (JL12668) were purchased from Shanghai Jianglai Biotechnology Co., Ltd. The biochemical assay kits for BUN (CO 13-2-1) and CRE (CO 11-2-1) were sourced from Nanjing Jiancheng Bioengineering Institute (Nanjing, China). Nitric acid (211020547, UP) and hydrogen peroxide (211026235, UP) were purchased from Jiangsu Crystal Clear Electronic Material Co., Ltd. Anhydrous ethanol (100092683, AR), xylene (10023418, AR) and neutral balsam (10,004,160) were obtained from Sinopharm Chemical Reagent Co., Ltd. An H&E staining kit (G1003) was purchased from Wuhan Servicebio Technology Co., Ltd., and an arsenic standard solution (100 μg/mL, GBW (E) 080117) was purchased from the National Institute of Metrology, China. A total of 54 female CD-1 mice, aged 4 weeks and weighing 20 ± 2 g, were purchased from Sipeifu (Beijing) Biotechnology Co., Ltd. The mice were housed in an SPF environment at 22°C ± 1°C with a light cycle from 7:00 to 19:00. All animal experiments were approved by the Ethics Committee of the Northwest Institute of Plateau Biology, Chinese Academy of Sciences (Approval No: NWIPB20171106-01), and followed the “Guide for the Care and Use of Laboratory Animals” (1978 revised edition) issued by the National Institutes of Health.

### 2.2 Preparation of wild *Cordyceps sinensis* powder, arsenic content, and arsenic speciation analysis

The soil adhering to the wild *C. sinensis* was removed, and the samples were washed thoroughly with ultrapure water. After drying in a 40°C oven, the samples were ground into powder and sieved through a 60-mesh sieve to obtain wild *C. sinensis* powder. In previous experiments, the total arsenic content and speciation in wild *C. sinensis* were determined via high-performance liquid chromatography coupled with atomic fluorescence spectrometry (HPLC-AFS). A standard calibration curve was constructed by analyzing standard solutions of varying concentrations, and after methodological validation, arsenic speciation in the samples was detected. The concentrations of different arsenic species were calculated via a linear equation. The total arsenic content in wild *C. sinensis* was found to be 16.36 mg/kg, with an arsenite to arsenate content ratio of 1:2. (Ma, 2024).

### 2.3 Grouping and administration

**FIGURE 1:**
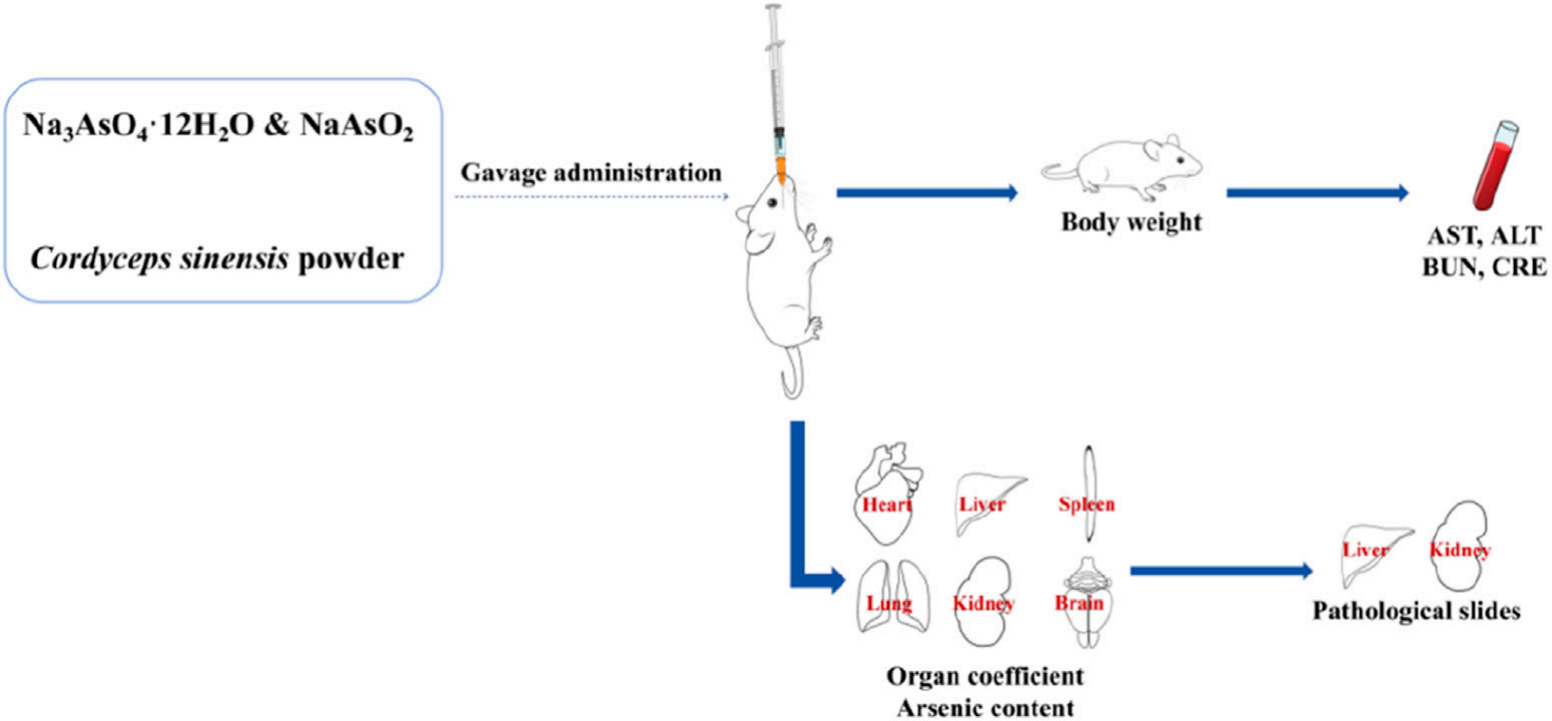
Schematic diagram of the experimental procedure.

The mice were randomly divided into three groups (*n* = 18 per group) on the basis of their body weight: the control group, the wild *C. sinensis* group (Cordyceps), and the inorganic arsenic group (arsenic). The control group was administered ultrapure water by gavage (0.1 mL/10 g). Wild *C. sinensis* group: Gavage with a wild *C. sinensis* suspension at a dose of 1.85 g/kg/day (the maximum dose recorded in the pharmacopoeia); arsenic group: Aqueous solution of arsenite and arsenate compounds, with a total arsenic dose of 0.03 mg/kg/day (equivalent to the total arsenic content found in the wild *C. sinensis* used in this study). The grouping treatment is shown in Table 1, and the experimental procedure is illustrated in Figure 1.

**TABLE 1:** Drug treatment for different groups.

Group	Treatment	Dosage	Total arsenic dose	Note
Control	Gavage with ultrapure water	0.1 mL/10 g	0 mg/kg	Not applicable
Cordyceps	Gavage with wild *Cordyceps sinensis*	1.85 g/kg/day	16.36 mg/kg	The arsenic content, the ratio of arsenite to arsenate in the arsenic group, is consistent with the arsenic content and the ratio of arsenite to arsenate in wild *Cordyceps sinensis*
Arsenic	Gavage with Inorganic arsine	0.03 mg/kg/day (arsenite: arsenate = 1:2)	16.36 mg/kg	

Conversion of administered doses was performed via the following formula: human equivalent dose (HED, mg/kg) = mouse dose (mg/kg) × mouse km/human km. Where the mouse km is three and the human km is 37 (Reagan-Shaw et al., 2008). Conversion of the wild *Cordyceps sinensis* dose for mice was performed with the following formula: 9 g/60 kg = mouse dose×3/37. For the arsenic compounds, the dose for the mice was calculated via the same formula: 0.147 mg/60 kg (the arsenic content in 9 g of wild *Cordyceps sinensis*) = Mice dose×3/37. The dose for the mice was 0.03 mg/kg. The dosage for the mice was 0.1 mL/10 g. To calculate the total mass of arsenic required for the preparation of 1 mL, 0.003 mg of arsenite:arsenate = 1:2 was used, and the following formula was used to determine the mass of arsenic compounds needed: molecular weight of arsenic/molecular weight of the arsenic compound = mass of arsenic/mass of the arsenic compound. The molecular weight of arsenic is 74.92, the molecular weight of sodium arsenite is 129.91, and the molecular weight of sodium arsenate dodecahydrate is 424.072. For sodium arsenite, the purity was 95%, and for sodium arsenate dodecahydrate, the purity was greater than 98%.

### 2.4 Body weight measurement

Body weight was measured weekly after a 12-h fast to monitor weight changes over the course of the study.

### 2.5 Euthanasia procedure

The mice were anesthetized via isoflurane inhalation to ensure that the procedures were painless. Once the mice were fully unconscious, a sterile disposable needle was used to perform direct cardiac puncture through the midline of the sternum to rapidly induce euthanasia and simultaneously collect undiluted blood samples. All procedures were conducted in accordance with national and institutional animal use and care guidelines, and all personnel received professional training.

### 2.6 Organ indices measurement

#### 2.6.1 Organ-to-body weight ratio

One, two, and 4 weeks after treatment, six mice from each group were anesthetized, and their hearts, livers, spleens, lungs, kidneys, and brains were collected and weighed to calculate the organ coefficients. The formula for calculating the organ-to-body weight ratio is as follows (Kluwe, 1981): organ-to-body weight ratio (%) = (organ weight/body weight) × 100.

#### 2.6.2 Organ-to-brain weight ratio

The hearts, livers, spleens, lungs, kidneys, and brains of the mice were collected and weighed to calculate the organ‒brain ratio. The formula for calculating the organ-to-brain weight ratio is as follows (Sellers et al., 2007): organ-to-brain weight ratio (%) = (organ weight/brain weight) × 100.

### 2.7 Arsenic content measurement

For arsenic content analysis, approximately 0.3 g (to 0.0001 g precision) of each organ (heart, liver, spleen, lung, kidney, and brain) was accurately weighed. The samples were then soaked overnight in 3 mL of nitric acid for preliminary digestion, followed by 2 mL of hydrogen peroxide for 2 h. The samples were subjected to microwave digestion and then evaporated on a hot plate at 120°C for 20 min. After cooling to room temperature, the samples were diluted with ultrapure water to a final volume of 50 mL and then ready for arsenic content measurement. The prepared samples were analyzed in standard mode, with each sample being tested in triplicate. Instrument conditions: RF power: 1150 W, plasma gas flow rate: 18 L/min, carrier gas flow rate: 0.65 L/min, auxiliary gas flow rate: 1.20 L/min, Ce++/Ce140 ratio: 0.019, Ce156/Ce140 ratio: 0.023.

### 2.8 Measurement of AST, ALT, BUN, and CRE levels in serum

One, two, and 4 weeks after treatment, six mice from each group were anesthetized, and blood was collected from their hearts. The blood was placed in clotting tubes, incubated at 4°C, and then centrifuged at 3000 rpm for 15 min to obtain the serum. The serum was transferred to 1.5 mL centrifuge tubes. The levels of AST and ALT in the serum were measured via ELISA kits, whereas the BUN and CRE levels were determined via biochemical assay kits.

### 2.9 H&E staining and pathological analysis

The left lobe of the liver and the left kidney of each mouse were fixed in 4% paraformaldehyde, embedded in paraffin, and sectioned into 5 μm thick slices. The slices were then subjected to routine hematoxylin and eosin (H&E) staining for pathological analysis. The scoring of hepatic and renal pathological damage is based on the standards proposed by Mann (Table 2), which classify the degree of damage into no damage (0 points), very mild damage (1 point), mild damage (2 points), moderate damage (3 points), and severe damage (4 points). Each tissue section was randomly selected from at least three different fields of view, and the scoring was conducted by two professionals via a blinded method (Mann, 2019).

**TABLE 2:** Hepatic and renal tissue pathology scoring criteria.

Level	Stage	Description
0	Normal	Tissue considered to be normal, under the conditions of the study and considering the age, sex, and strainof the animal concerned. Alterations may be present, which, under other circumstances, would beconsidered deviations from normal
1	Minimal	The amount of change present barely exceeds that which is considered to be within normal limits
2	Slight	In general, the lesion is easily identified but of limited severity
3	Moderate	The lesion is prominent, but there is significant potential for increased severity
4	Severe	The degree of change is as complete as possible (occupies the majority of the organ)

### 2.10 Statistical analysis

The data were analyzed, and graphs were generated via GraphPad Prism 10.0 software, with the results expressed as the means ± SEMs. Two-way analysis of variance (ANOVA) was used to assess differences between groups. Statistical significance was determined as follows: *p < 0.05, **p < 0.01, ***p < 0.001, and ****p < 0.0001.

## 3 Results

### 3.1 Neither wild *Cordyceps sinensis* nor inorganic arsenic significantly affected body weight changes in mice

**FIGURE 2:**
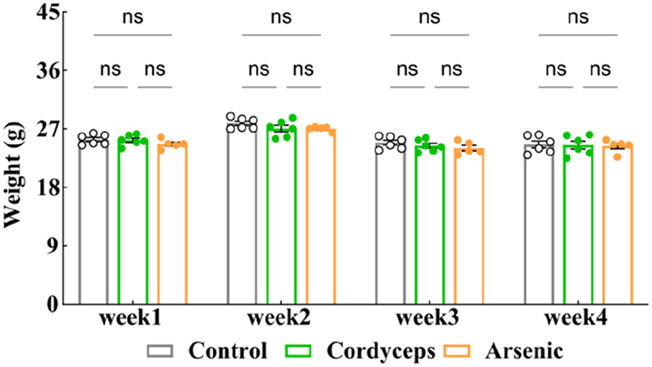
Mouse body weight changes. Note: *n* = 6.

Changes in body weight are among the commonly used physiological indicators for assessing the impact of a drug on mouse health and can be utilized to evaluate potential toxicological responses (Sellers et al., 2007). Because the inorganic arsenic present in wild *Cordyceps sinensis* (*C. sinensis*) may affect weight changes in mice, to investigate the differences in the influence of wild *C. sinensis* containing inorganic arsenic *versus* an equivalent dose of inorganic arsenic alone on body weight in mice, we monitored changes in body weight during the administration period. The results revealed that the weight changes in the three groups of mice followed a similar trend (Figure 2). The body weights of the mice in the Cordyceps group decreased by 0.004%, 0.029%, 0.019%, and 0.004% in the first, second, third, and fourth weeks of administration, respectively. In the arsenic group, the body weight decreased by 0.025%, 0.028%, 0.029%, and 0.011% at the first, second, third, and fourth weeks, respectively (Fig. S-1). No statistically significant differences in body weight changes were observed between the two groups and the control group. The experimental results indicate that treatment with wild *C. sinensis* had no impact on mouse body weight. Similarly, exposure to inorganic arsenic at equivalent doses had no influence on mouse body weight.

### 3.2 Wild *Cordyceps sinensis* has a far lower influence on organ indices than an equivalent dose of inorganic arsenic

#### 3.2.1 Wild *Cordyceps sinensis* had a lower influence on the organ-to-body weight ratio than an equivalent dose of inorganic arsenic

**FIGURE 3:**
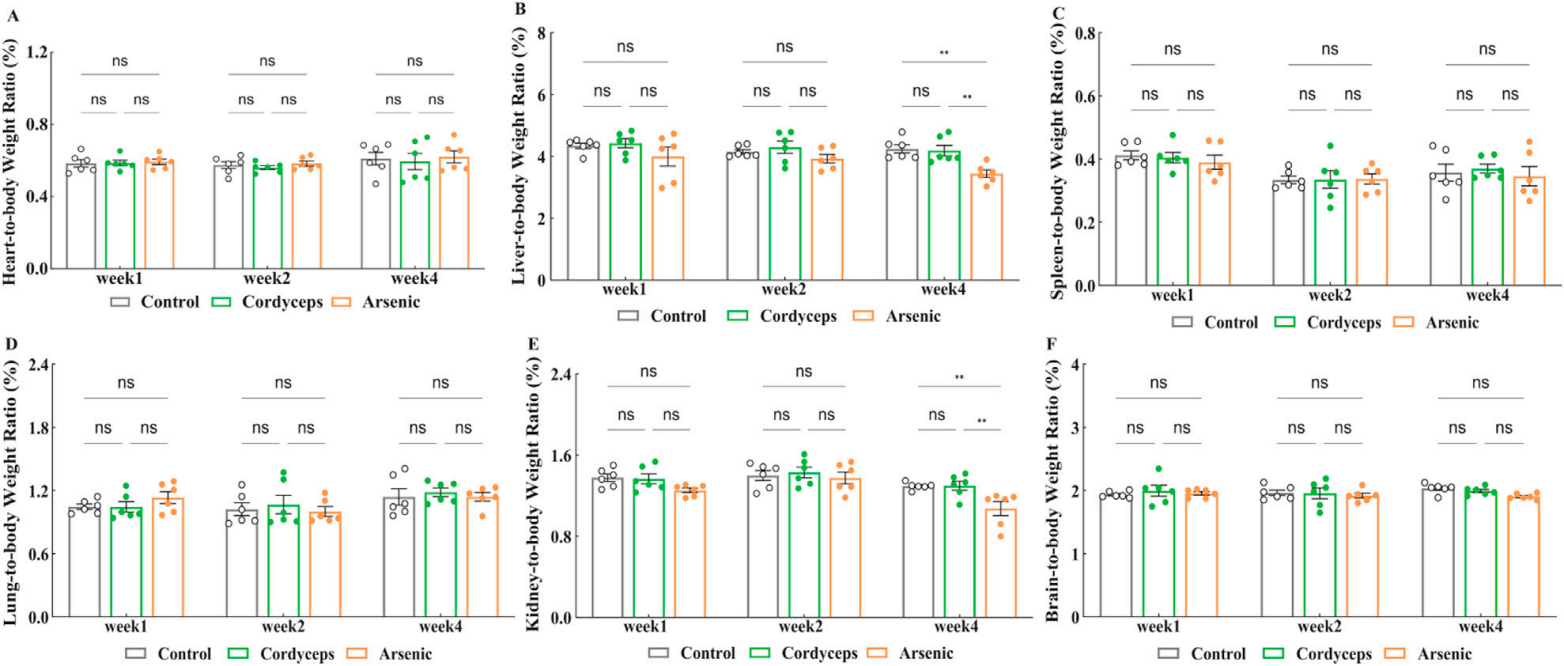
Changes in the organ-to-body weight ratio. **(A)** Heart-to-body weight ratio after treatment; **(B)** Liver-to-body weight ratio after treatment; **(C)** Spleen-to-body weight ratio after treatment; **(D)** Lung-to-body weight ratio after treatment; **(E)** Kidney-to-body weight ratio after treatment; **(F)** Brain-to-body weight ratio after treatment. Note: *n* = 6.

No significant differences in body weight changes were observed among the mice during the treatment period. However, changes in the organ-to-body weight ratio are more effective at reflecting organ damage induced by arsenic exposure (Abernathy et al., 1999). To evaluate the impacts of wild *Cordyceps sinensis* containing inorganic arsenic *versus* an equivalent dose of inorganic arsenic alone, we examined the organ‒to‒body weight ratios in mice. The results revealed no significant changes in the organ‒to‒body weight ratios of the heart, liver, spleen, lungs, kidneys, and brain in the Cordyceps group (Figure 3A‒F). Compared with those in the control group, the organ-to-body weight ratios of the liver and kidneys in the arsenic group significantly decreased at the fourth week (Arsenic vs. Control: 3.43 ± 0.11 vs. 4.24 ± 0.13, (liver organ-to-body weight ratio), *n = 6*, *p* < 0.01; Arsenic vs. Control: 1.07 ± 0.06 vs. 1.29 ± 0.01, (kidney organ-to-body weight ratio), *n = 6*, *p* < 0.01; Figures 3B,E), with a greater reduction observed over time (Fig. S-2). Therefore, wild *C. sinensis* treatment did not affect the organ-to-body weight ratio in mice, whereas inorganic arsenic exposure may lead to liver and kidney atrophy.

#### 3.2.2 Wild *Cordyceps sinensis* had a lower impact on the organ-to-brain weight ratio compared to an equivalent dose of inorganic arsenic

**FIGURE 4:**
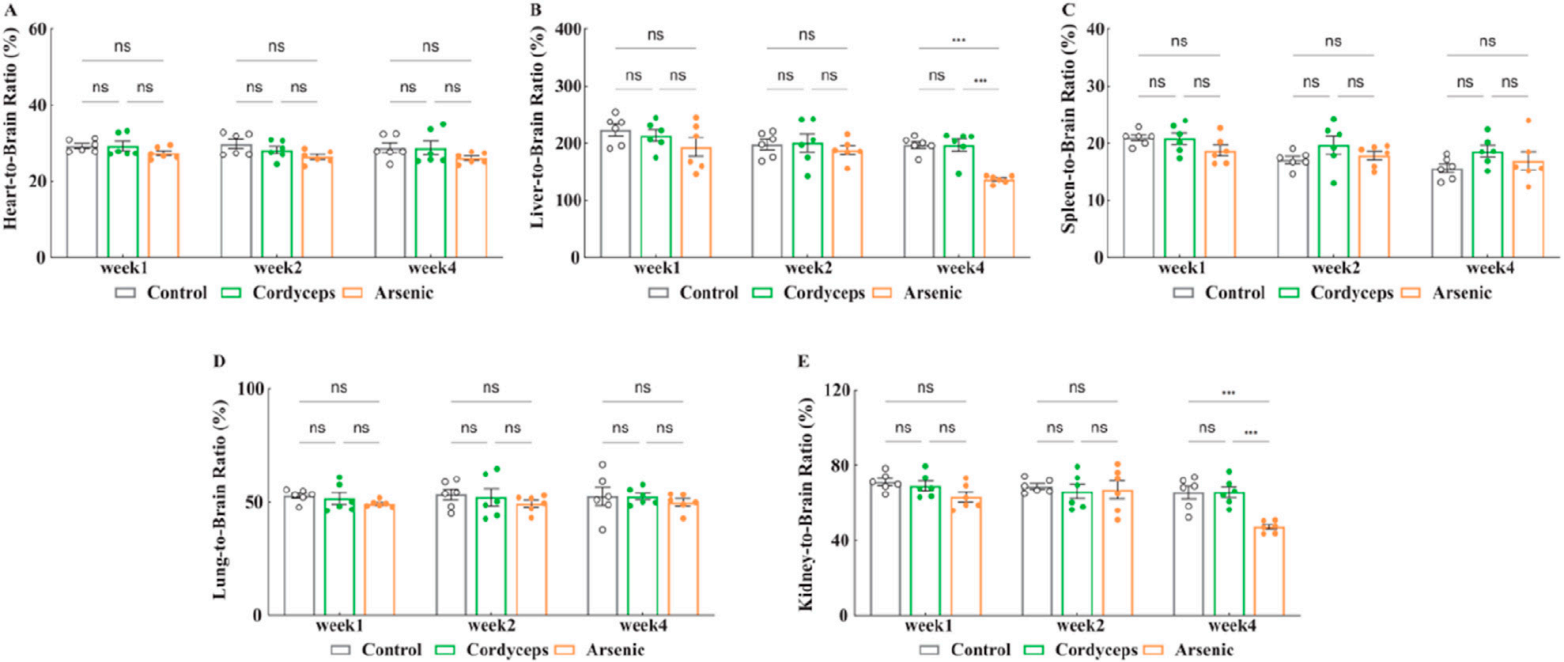
Changes in the organ-to-brain weight ratio. **(A)** Heart-to-brain weight ratio after treatment; **(B)** Liver-to-brain weight ratio after treatment; **(C)** Spleen-to-brain weight ratio after treatment; **(D)** Lung-to-brain weight ratio after treatment; **(E)** Kidney-to-brain weight ratio after treatment. Note: *n* = 6.

Next, we assessed the organ-to-brain weight ratios, which are less influenced by weight fluctuations and are considered a more objective reflection of organ changes (Bailey et al., 2004; Verhaegen and Van Gaal, 2019). The results revealed no significant changes in the organ‒to‒brain weight ratios of the heart, liver, spleen, lungs, and kidneys in the Cordyceps group (Figure 4A–E). Compared with those in the control group, the organ‒brain weight ratios of the liver and kidneys significantly decreased in the arsenic group at the fourth week (Arsenic vs. Control: 136.62 ± 2.65 vs. 196.79 ± 5.81, (liver organ‒brain weight ratio), *n = 6*, *p* < 0.01; Arsenic vs. Control: 47.25 ± 1.30 vs. 65.57 ± 3.42, (kidney organ‒brain weight ratio), *n = 6*, *p* < 0.01; Figures 4B,E), which is consistent with the trends observed in the organ‒to‒body weight ratios (Fig. S-3). These findings indicate that wild *Cordyceps sinensis* has no significant influence on the organ-to-brain weight ratio; however, inorganic arsenic exposure leads to atrophy of the liver and kidneys. The organ-to-body and organ-to-brain weight ratios were consistent, indicating that wild *C. sinensis* treatment did not significantly impact these ratios in mice. In contrast, inorganic arsenic exposure significantly reduced the liver and kidney ratios, indicating that inorganic arsenic-induced atrophy may impair organ function.

### 3.3 Wild *Cordyceps sinensis* had a far lower arsenic accumulation in mice compared to equivalent dose of inorganic arsenic

**FIGURE 5:**
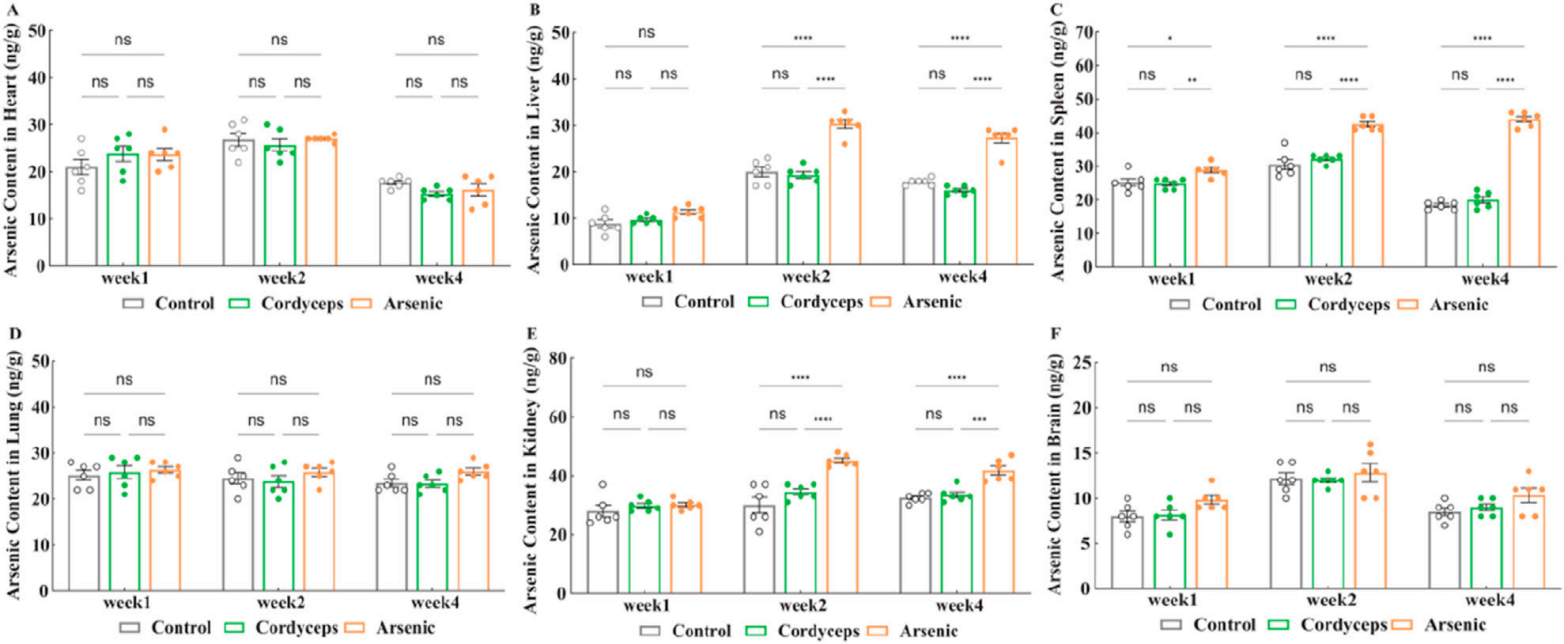
Changes of arsenic content in organs. **(A)** Arsenic content in the heart after treatment; **(B)** Arsenic content in the liver after treatment; **(C)** Arsenic content in the spleen after treatment; **(D)** Arsenic content in the lung after treatment; **(E)** Arsenic content in the kidney after treatment; **(F)** Arsenic content in the brain after treatment. Note: *n* = 6.

Our preliminary studies indicated that inorganic arsenic induced atrophy in the liver and kidneys of mice. Moreover, the accumulation of inorganic arsenic in the body is the basis of its toxic impacts. Prolonged inorganic arsenic exposure could lead to its accumulation in organs such as the liver and kidneys, resulting in damage to these organs (Abdul et al., 2015). To investigate the differences in organ accumulation between wild *Cordyceps sinensis* and an equivalent dose of inorganic arsenic in mice, we systematically measured the arsenic content in various organs. In the Cordyceps group, no significant differences were detected in the arsenic levels in the heart, liver, spleen, lungs, kidneys, and brain (Figures 5A–F). In the arsenic group, the spleen arsenic levels continuously increased during the treatment period and were significantly higher than those in the other two groups (*p* < 0.01, Figure 5C). The Arsenic levels in the liver and kidneys peaked in the second week and decreased by the fourth week, but the arsenic content in these organs remained significantly higher than that in the other two groups (*p* < 0.01, Figures 5B,E, Supplementary Figure S4), and no significant changes were observed in the arsenic levels in the other organs (Figures 5A,C,D,F). Moreover, compared with the Cordyceps group, the arsenic group presented significantly higher arsenic contents in the liver, kidneys, and spleen (*p* < 0.01, Figures 5B,C,E). In summary, no arsenic accumulation was detected in the organs of the mice treated with wild *C. sinensis*; conversely, the arsenic accumulation in the livers, kidneys, and spleens of the mice in the arsenic group was noticeable.

### 3.4 Wild *Cordyceps sinensis* had a far lower impact on serum AST and ALT and liver histopathology compared to an equivalent dose of inorganic arsenic

**FIGURE 6:**
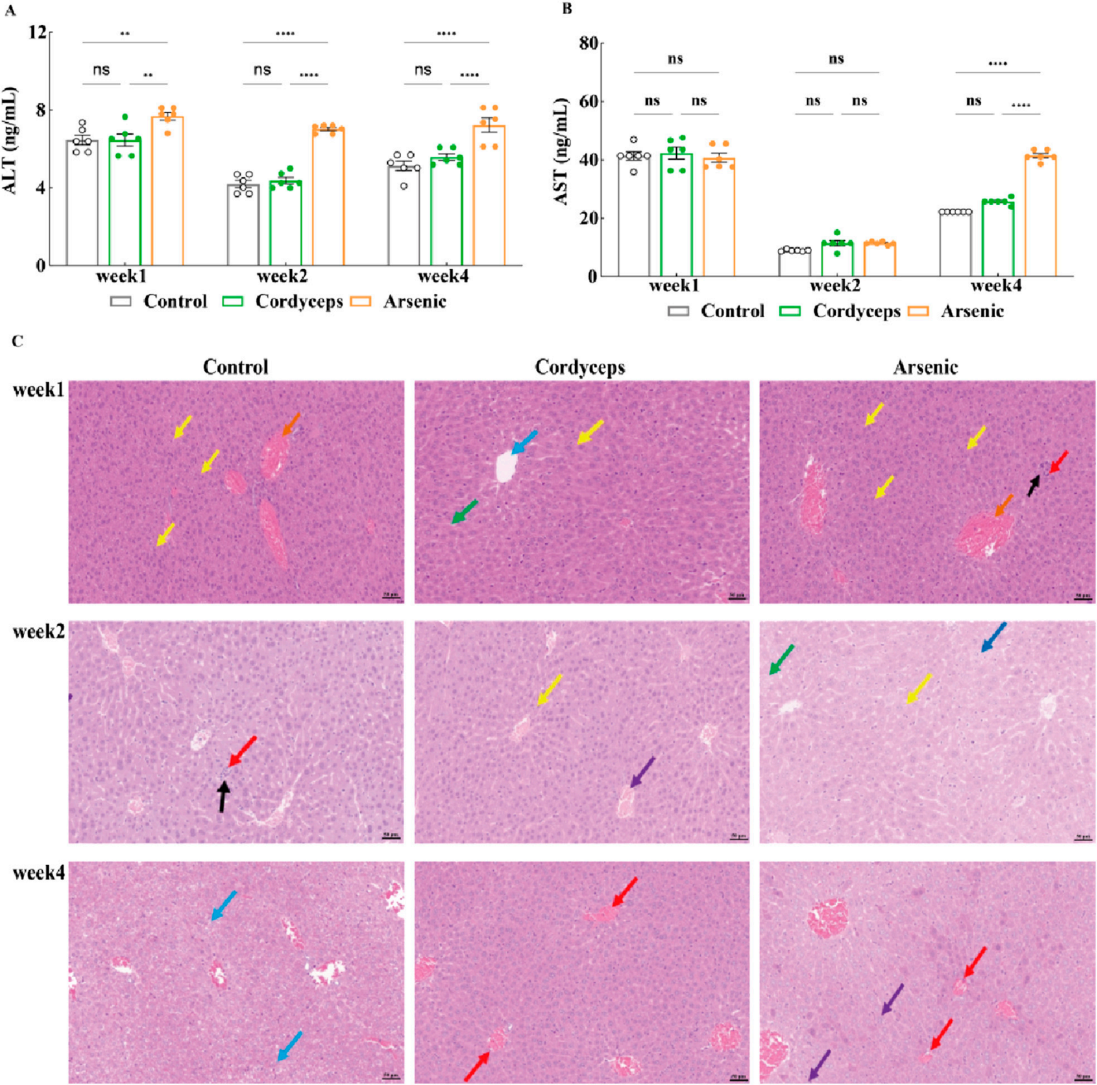
Changes in the serum AST and ALT levels and pathological sections of the liver. **(A)** Serum ALT levels in the mice; **(B)** Serum AST levels in the mice; **(C)** Histopathological sections of the mouse liver (20×). Note: For the AST and ALT tests, the sample size was *n = 6*, and for the H&E analysis, the sample size was *n = 3*. In the figures above, blue arrows indicate ballooning degeneration of liver cells; yellow arrows denote mild fatty degeneration of liver cells; black arrows indicate focal necrosis of liver cells; dark blue arrows highlight vacuolar degeneration of liver cells; red arrows indicate scattered lymphocyte infiltration in the liver; and purple arrows indicate hepatic vascular congestion.

We have found that the mixture of inorganic arsenic compounds at a total arsenic dose equivalent to that in wild Cordyceps sinensis, significantly altered renal organ indices and resulted in marked arsenic accumulation in the kidneys, suggesting potential nephrotoxic impacts of inorganic arsenic. Serum biochemical markers of liver function (AST and ALT) and liver histopathological sections more accurately reflect the extent of hepatic injury (Gerstein et al., 2014). To investigate the impact of wild *C. sinensis* and equivalent doses of inorganic arsenic on the liver in mice, we further assessed serum biochemical markers of liver function and performed histopathological examination of liver tissues. The results revealed that the serum levels of AST and ALT in the Cordyceps group did not significantly differ from those in the control group (Figures 6A,B). In contrast, the arsenic group presented a marked increase in AST and ALT levels, which peaked at week two and declined by week 4, although they remained significantly higher than those in the other groups (p < 0.01; Figures 6A,B, Supplementary Figure S5). Histopathology confirmed these biochemical changes: the Cordyceps group maintained intact liver architecture, with round and plump hepatocytes and no evident pathological changes (Figure 6C). In the arsenic-exposed mice, however, progressive liver damage, including mild fatty degeneration, hydropic degeneration, and focal necrosis at early stages, followed by lymphocytic infiltration and ballooning degeneration by week 4, was observed (Figure 6C). These observations were confirmed by liver histopathological scoring, which revealed no significant changes in the Cordyceps group but revealed progressively worsening inflammatory, necrotic, and degenerative changes in the arsenic group (p < 0.05; Table 3). Taken together, these results demonstrate that wild *C. sinensis* intervention did not adversely affect liver function or histology, whereas arsenic exposure induced cumulative hepatotoxicity, as evidenced by biochemical, structural, and scoring analyses.

**TABLE 3:** Hepatic lesion pathology scoring.

Time period	Group	Case count	Total histopathological score	Inflammatory cell infiltration	Necrosis	Degeneration	*p* value
Week1	Control	3	0	0	0	0	*p* > 0.05
	Cordyceps	3	1	1	0	0	*p* > 0.05
	Arsenic	3	3	1	1	1	*p* < 0.05
Week2	Control	3	1	1	0	0	*p* > 0.05
	Cordyceps	3	1	0	1	0	*p* > 0.05
	Arsenic	3	2	1	1	0	*p* > 0.05
Week4	Control	3	0	0	0	0	*p* > 0.05
	Cordyceps	3	1	1	0	0	*p* > 0.05
	Arsenic	3	3	0	0	1	*p* < 0.05

### 3.5 Wild *Cordyceps sinensis* has a far lower impact on serum BUN and CRE and renal histopathology compared to an equivalent dose of inorganic arsenic

**FIGURE 7:**
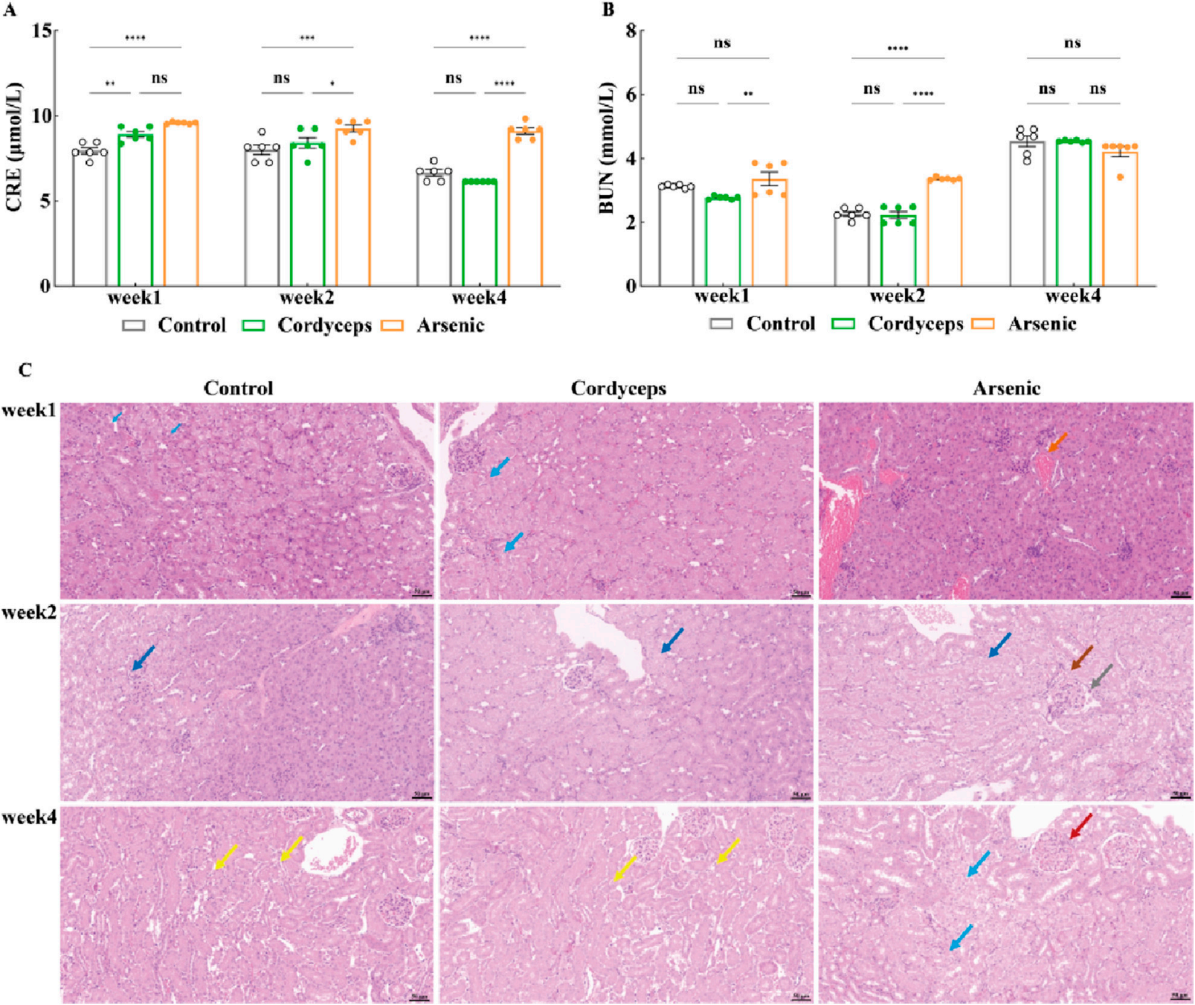
Changes in serum CRE and BUN levels and pathological kidney sections. **(A)** Serum CRE levels in mice; **(B)** Serum BUN levels in mice; **(C)** Histopathological sections of mouse kidneys (20×). Note: For the BUN and CRE tests, the sample size was *n = 6*, and for the H&E analysis, the sample size was *n = 3*. In the figures above, blue arrows indicate renal tubular epithelial cell vacuolar degeneration; yellow arrows denote the uniform distribution of glomeruli in the kidney; green arrows represent mild dilation of renal tubules; dark blue arrows highlight edema of renal tubular epithelial cells; and orange arrows represent minimal vascular congestion in the kidney interstitium.

Our preliminary findings revealed that inorganic arsenic, at a dose equivalent to that found in wild *Cordyceps sinensis*, significantly altered renal organ indices and resulted in marked arsenic accumulation in the kidneys, suggesting potential nephrotoxic impacts of inorganic arsenic. Serum biochemical markers of renal function (BUN, CRE) and renal histopathological data can be used to assess the degree of kidney injury more precisely (Bonventre et al., 2010). Therefore, to further investigate the impact of wild *C. sinensis* and equivalent total arsenic dose of inorganic arsenic compounds mixture on mice renal function, we assessed serum biochemical markers of kidney function and examined histopathological changes in renal tissue. Serum biochemical analysis revealed that serum CRE in the Cordyceps group were significantly elevated at week 1 (P < 0.01), but showed no significant difference at weeks 2 and 4. In contrast, serum BUN remained unchanged throughout the dosing period (Figures 7A, B). In the arsenic group, the serum CRE and BUN levels were significantly elevated (*p* < 0.01, Figures 7A,B), peaked at week 2, and gradually decreased by week four but were still significantly higher than those in the other groups (*p* < 0.01, Figures 7A,B, Supplementary Figure S6). These biochemical alterations were corroborated by histopathological findings. Renal histology in the Cordyceps group mice maintained intact kidney architecture, characterized by uniformly distributed glomerular cells and matrixes, without interstitial proliferation and immune cells infiltration (Figure 7C). In contrast, inorganic arsenic exposure caused congestion in the renal interstitial vasculature, eosinophilic granulocyte aggregation in the glomerular capsule, and at week 4, edema-like degeneration of renal tubular epithelial cells, vascular congestion in the interstitium, and eosinophilic granulocyte accumulation were observed (Figure 7C). Quantitative histopathological scoring further validated these observations. The Cordyceps group of mice is shown in Table 4. In contrast, the arsenic group exhibited higher pathology scores for hydropic degeneration and inflammatory cell infiltration at both week two and week four than the control and Cordyceps groups did (Table 4). In summary, wild *C. sinensis* did not significantly influence kidney function in mice, whereas arsenic exposure significantly impaired renal excretory function and caused structural damage to kidney tissue.

**TABLE 4:** Renal lesion pathology scoring.

Time period	Group	Case count	Total histopathological score	Hydropic degeneration	Vacuolar degeneration	Dilation	Inflammatory cell infiltration	*p* value
Week1	Control	3	1	1	0	0	0	*p* > 0.05
	Cordyceps	3	1	1	0	0	1	*p* > 0.05
	Arsenic	3	2	1	0	0	1	*p* > 0.05
Week2	Control	3	1	1	0	0	0	*p* > 0.05
	Cordyceps	3	1	1	0	0	0	*p* > 0.05
	Arsenic	3	3	3	0	0	0	*p* > 0.05
Week4	Control	3	1	1	0	0	0	*p* > 0.05
	Cordyceps	3	1	1	0	0	0	*p* > 0.05
	Arsenic	3	2	1	0	0	1	*p* > 0.05

## 4 Discussion

This study assessed the hepatorenal toxicity of wild *Cordyceps sinensis* (*C. sinensis*) at its maximum clinical dose and compared it with equivalent total arsenic dose of inorganic arsenic compounds mixture (sodium arsenite and sodium arsenate). The results demonstrate that administering wild *C. sinensis* at the maximum clinical dose does not produce significant changes in organ coefficients, tissue arsenic levels, serum biomarkers of liver function, and liver histopathological morphology in mice. Furthermore, no significant alterations were observed in serum biomarkers of renal function at weeks 2 and 4, nor in renal histopathology throughout the treatment period. In contrast, equivalent doses of inorganic arsenic compounds significantly reduced organ-to-body weight ratios, increased arsenic accumulation, impaired liver and kidney function, and induced pronounced tissue damage. Collectively, these findings indicate that under the tested conditions, wild *C. sinensis* does not elicit hepatorenal toxicity comparable to that of inorganic arsenic compounds, providing novel evidence supporting its safety.

At the systemic level, the stable body weight and organ-to-body weight ratios observed in the *C. sinensis* treatment group underscore its minimal toxicity. In contrast, inorganic arsenic exposure led to significant decreases in both body weight and organ-to-body weight ratios, particularly affecting liver and kidney mass. This finding aligns with Basher et al., who reported reduced kidney and heart weights following prenatal arsenic exposure (Basher et al., 2023), and with Lai et al., who reported significant decreases in body weight and liver mass after chronic inorganic arsenic administration (Lai et al., 2024). The reduction in organ mass likely stems from oxidative stress-induced cellular damage. Hughes and Xie et al. demonstrated that inorganic arsenic exacerbates reactive oxygen species (ROS) production, leading to excess free radical accumulation. This oxidative process damages cell membranes, proteins, and DNA and ultimately triggers apoptosis and necrosis, reducing the cellular density and organ mass (Hughes, 2002; Xie et al., 2004). Thus, wild *C. sinensis* showed no systemic toxicity under the tested conditions, whereas inorganic arsenic-induced toxicity clearly correlated with tissue-specific arsenic accumulation.

The quantification of arsenic levels in target organs further validated the pivotal role of tissue-specific arsenic accumulation in mediating toxicity. The Cordyceps group presented undetectable arsenic levels in liver and kidney tissues, indicating minimal arsenic burden. In contrast, the arsenic group presented substantial arsenic accumulation, supporting the hypothesis that tissue accumulation determines arsenic toxicity (Naujokas et al., 2013). Our findings align with those of Xie et al. and Flora et al., who reported dose-dependent arsenic accumulation in hepatic and renal tissues (Flora et al., 1997; Xie et al., 2004). Therefore, arsenic-induced hepatorenal toxicity depends on tissue accumulation, whereas the extremely low arsenic levels observed in the Cordyceps group suggest that under the tested conditions, the accumulation risk is negligible.

Furthermore, comparing the dosing employed in this study with international regulatory standards enhances the toxicological relevance of our findings. According to the International Council for Harmonization of Technical Requirements for Pharmaceuticals for Human Use (ICH) guideline Q3D, the permissible daily exposure (PDE) for arsenic is set at 15 μg/day, with a No-Observed-Adverse-Effect Level (NOAEL) of 0.3 μg/kg/day (ICH, 2022). Similarly, the U.S. Environmental Protection Agency (EPA) has set an oral Reference Dose (RfD) for inorganic arsenic at 0.3 μg/kg/day (EPA, 2017), and the Agency for Toxic Substances and Disease Registry (ATSDR) defines the Minimal Risk Level (MRL) for intermediate (15–364 days) and long-term (≥365 days) inorganic arsenic exposure at the same level (0.3 μg/kg/day) (ATSDR, 2007), which is equivalent to a mouse dose of 3.69 μg/kg/day. In this study, the total arsenic content of wild *C. sinensis* was 16.36 mg/kg, corresponding to an equivalent murine dose of 0.03 mg/kg. This exceeds internationally established limits and is therefore predicted to lead to arsenic accumulation in mice. Nevertheless, 4-week organ--specific analysis revealed no significant increase in arsenic concentrations in either liver or kidney tissues in the Cordyceps group. In contrast, mice receiving the same dose of inorganic arsenic accumulated 26 ± 5 ng/g in the liver and 38 ± 4 ng/g in the kidneys. Xi et al. demonstrated that hepatic arsenic burdens remain below 100 ng/g under threshold exposure conditions (Xi et al., 2010), and Jomova et al. similarly reported minimal organ accumulation in populations complying with regulatory standards (Jomova et al., 2015). In summary, these data further underscore the favorable safety profile of wild *C. sinensis* under the tested conditions, as no arsenic accumulation in organs was observed even at exposure levels exceeding international standards.

Further analyses, including serum biochemical markers and histopathological examinations, clearly revealed the toxicological differences between wild *C. sinensis* and equivalent doses of inorganic arsenic. The administration of wild *C. sinensis* resulted in no significant hepatic or renal injury, whereas inorganic arsenic exposure significantly increased the serum alanine aminotransferase (ALT) and aspartate aminotransferase (AST) levels, which was accompanied by liver pathology characterized by hepatocellular necrosis, steatosis, and inflammatory infiltration. These outcomes are consistent with those of Flora and Liu et al., who reported that oxidative stress and mitochondrial dysfunction are critical arsenic-induced hepatotoxic mechanisms (Flora et al., 1997; Liu and Waalkes, 2008). Additionally, the arsenic in wild *C. sinensis* may be metabolized in the liver through arsenic methyltransferase (As3MT). This mechanism is consistent with findings from studies on the Realgar-Indigo naturalis formula (RIF), which modulates the activity of CYP450 enzymes and the expression of As3MT, influencing arsenic methylation metabolism and thereby reducing its toxicity (Jiang et al., 2023; Xu et al., 2017). Renal toxicity is manifested by elevated blood urea nitrogen (BUN) and creatinine (CRE) levels, as well as histopathological damage to the renal tubules, which was observed only in the mice treated with inorganic arsenic, corroborating previously described oxidative stress-mediated renal toxicity (Flora, 2011; Liu et al., 2001; Xu et al., 2017). Collectively, these results demonstrate that under the tested conditions, the hepatorenal toxicity of wild *C. sinensis* is superior to that of equivalent doses of inorganic arsenic. This effect may be attributable to the hepatoprotective and nephroprotective activities of bioactive constituents in wild *C. sinensis*, such as polysaccharides and H1A-type sterols (Lin and Li, 2011).

Despite these promising outcomes, our study is limited by its 4-week duration, which reflects only medium-term toxicological responses. Long-term safety data and chronic exposure studies of wild *C. sinensis* are necessary. Additionally, our control group comprised inorganic arsenic compounds with equivalent arsenic contents but greater intrinsic toxicity, potentially limiting direct comparisons. Further speciation analyses of arsenic in wild *C. sinensis* are warranted to elucidate the distinct biological roles and toxicities of its organic arsenicals. Moreover, subsequent experiments will consider adding a male control group and increasing the sample size to enhance the generalizability of the data. Finally, while our data suggest that oxidative stress is a plausible mechanism underlying arsenic-induced hepatorenal injury, this study did not directly assess oxidative markers, and further mechanistic studies are needed for a comprehensive understanding.

## 5 Conclusion

In this study, we assessed the relative safety of wild *Cordyceps sinensis (C. sinensis)* by comparing its hepatorenal toxicity with that of a mixture of inorganic arsenic compounds (sodium arsenate and sodium arsenite) administered at an equivalent total arsenic dose. Despite containing inorganic arsenic, wild *C. sinensis* did not induce significant arsenic accumulation in major organs, including the heart, liver, spleen, lung, kidney, and brain, nor did it cause notable hepatorenal toxicity at its maximum clinical dose in long-term. In contrast, the inorganic arsenic compounds mixture led to marked arsenic accumulation in the liver and kidney, accompanied by evident organ dysfunction. These findings demonstrate that wild *C. sinensis*, when used at its clinically recommended dose, exhibits substantially lower toxicity and tissue arsenic burden compared to equivalent dose of inorganic arsenic compounds mixture, providing important evidence to support its relative safety.

## Data Availability

The original contributions presented in the study are included in the article/Supplementary Material, further inquiries can be directed to the corresponding authors.
