# Correction: Wild *Cordyceps sinensis* exhibits far lower arsenic accumulation and hepatorenal toxicity in mice compared to equivalent dose of inorganic arsenic

**DOI:** 10.3389/fphar.2025.1730109

**Published:** 2025-11-24

**Authors:** Liang Gao, Hongxia Yang, Jinmei Ma, Hongtao Bi, Yuancan Xiao, Cen Li, Lixin Wei

**Affiliations:** 1 Qinghai Provincial Key Laboratory of Tibetan Medicine Pharmacology and Safety Evaluation, Northwest Institute of Plateau Biology, Chinese Academy of Science, Xining, China; 2 CAS Key Laboratory of Tibetan Medicine Research, Northwest Institute of Plateau Biology, Chinese Academy of Sciences, Xining, China; 3 University of Chinese Academy of Sciences, Beijing, China; 4 Department of Pathobiology, School of Veterinary Medicine, University of Pennsylvania, Philadelphia, PA, United States

**Keywords:** wild cordyceps sinensis, inorganic arsenic, safety, arsenic accumulation, liver and kidney damage

The **title** of this article was erroneously given as: “Wild *Cordyceps sinensis* exhibits far lower arsenic accumulation and hepatorenal in mice compared to equivalent dose of inorganic arsenic”. The word “toxicity” was omitted. The correct title of the article is “Wild *Cordyceps sinensis* exhibits far lower arsenic accumulation and hepatorenal toxicity in mice compared to equivalent dose of inorganic arsenic”.

Throughout the published article, the authors have made revisions to the text, reworking and expanding on the contents.

In the abstract, the “**Conclusion**” was erroneously given as “Discussion”. The contents of the “Conclusion” have been revised.

A correction has been made to the **Abstract** section Paragraph Number4. The paragraph previously stated:

“Discussion: These findings indicate that wild Cordyceps sinensis exhibits no detectable toxicity even at arsenic exposure levels comparable with those of inorganic arsenic. This study provides critical experimental evidence supporting the safe consumption of wild Cordyceps sinensis.”

The corrected sentence appears below:

“Conclusion: These findings indicate that, at the maximum clinical dose, wild *Cordyceps sinensis* does not cause measurable hepatorenal toxicity in long-term and exhibits markedly greater safety compared to a mixture of inorganic arsenic compounds (sodium arsenate and sodium arsenite) at an equivalent total arsenic dose.”

A correction has been made to the section **Introduction**, Paragraph Number 4. This sentence previously stated:

“Collectively, these comparative data provide robust evidence that wild *C. sinensis* does not elicit detectable toxicity at clinically relevant exposure levels, thereby supporting its safe dietary use.”

The corrected sentence appears below:

“Collectively, these comparative data demonstrate that wild *C. sinensis* causes no detectable hepatorenal toxicity in mice at the maximum clinical dose in long-term and shows substantially safer than an equivalent total arsenic dose of inorganic arsenic compounds mixture (sodium arsenate and sodium arsenite), providing reference evidence for understanding its dietary safety.”

A correction has been made to the section 3 **Results**, 3.4 Wild *Cordyceps sinensis* had a far lower impact on serum AST and ALT and liver histopathology compared to an equivalent dose of inorganic arsenic, Paragraph Number 1. This sentence previously stated:

“Our preliminary findings revealed that inorganic arsenic, at a dose equivalent to that found in wild *Cordyceps sinensis*, significantly altered liver organ indices and resulted in marked arsenic accumulation in the liver, suggesting potential hepatotoxic impacts of inorganic arsenic.”

The corrected sentence appears below:

“We have found that the mixture of inorganic arsenic compounds at a total arsenic dose equivalent to that in wild *Cordyceps sinensis*, significantly altered renal organ indices and resulted in marked arsenic accumulation in the kidneys, suggesting potential nephrotoxic impacts of inorganic arsenic.”

A correction has been made to the section 3 **Results**, 3.5 Wild Cordyceps sinensis has a far lower impact on serum BUN and CRE and renal histopathology compared to an equivalent dose of inorganic arsenic, Paragraph Number 1. These sentences previously stated:

“To further investigate the impact of wild *C. sinensis* and equivalent doses of inorganic arsenic on renal function in mice, we assessed serum biochemical markers of kidney function and examined histopathological changes in renal tissues. Serum biochemical analysis revealed that there were no significant changes in the serum CRE and BUN levels in the Cordyceps group during the treatment period (Figures 7A,B).”

The corrected sentences appear below:

“Therefore, to further investigate the impact of wild *C. sinensis* and equivalent total arsenic dose of inorganic arsenic compounds mixture on mice renal function, we assessed serum biochemical markers of kidney function and examined histopathological changes in renal tissue. Serum biochemical analysis revealed that serum CRE in the Cordyceps group were significantly elevated at week 1 (P < 0.01), but showed no significant difference at weeks 2 and 4. In contrast, serum BUN remained unchanged throughout the dosing period (Figures 7A,B).”

A correction has been made to the section 3 **Results**, 3.5 Wild Cordyceps sinensis has a far lower impact on serum BUN and CRE and renal histopathology compared to an equivalent dose of inorganic arsenic, Paragraph Number 1. This sentence previously stated:

“The Cordyceps group was intact, with a uniform distribution of glomerular cells and matrix and no interstitial proliferation or inflammatory cell infiltration (Figure 7C).”

The corrected sentence appears below:

“Renal histology in the Cordyceps group mice maintained intact kidney architecture, characterized by uniformly distributed glomerular cells and matrixes, without interstitial proliferation and immune cells infiltration (Figure 7C).”

A correction has been made to the section 4 **Discussion**, Paragraph Number 1. These sentences previously stated:

“This study assessed the hepatotoxic and nephrotoxic potential of wild *Cordyceps sinensis* (*C. sinensis*) at its maximum clinical dose and compared it with equivalent doses of more toxic inorganic arsenic compounds-sodium arsenite and sodium arsenate, to comprehensively evaluate its safety. The results demonstrated that wild *C. sinensis* at the maximum clinical dose caused no significant changes in organ-to-body weight ratios, tissue arsenic levels, serum biomarkers of hepatic/renal function, or histopathological morphology in mice.”

The corrected sentences appear below:

“This study assessed the hepatorenal toxicity of wild *Cordyceps sinensis* (*C. sinensis*) at its maximum clinical dose and compared it with equivalent total arsenic dose of inorganic arsenic compounds mixture (sodium arsenite and sodium arsenate). The results demonstrate that administering wild *C. sinensis* at the maximum clinical dose does not produce significant changes in organ coefficients, tissue arsenic levels, serum biomarkers of liver function, and liver histopathological morphology in mice. Furthermore, no significant alterations were observed in serum biomarkers of renal function at weeks 2 and 4, nor in renal histopathology throughout the treatment period.”

A correction has been made to the section 5 **Conclusion**, Paragraph Number 1. This paragraph previously stated:

“In this study, we evaluated the safety of wild *C. sinensis* (*C. sinensis*) by comparing the impacts of total arsenic administered at the maximum clinical dose and an equivalent dose of inorganic arsenic compounds on murine hepatic and renal tissues. Our results demonstrated that, despite the presence of inorganic arsenic in wild *C. sinensis*, no significant tissue arsenic accumulation or hepatorenal toxicity was observed. Conversely, mice receiving equivalent doses of inorganic arsenic exhibited marked arsenic accumulation in hepatic and renal tissues, resulting in significant dysfunction. These findings confirm the relative safety of wild *C. sinensis* at clinical doses and underscore the pivotal role of tissue-specific arsenic accumulation in mediating toxicity. Overall, under equivalent arsenic dosing conditions, unlike inorganic arsenic compounds, wild *C. sinensis* did not induce histopathological or functional injury in liver or kidney tissues, demonstrating its favorable safety profile for consumption.”

The corrected paragraph appears below:

“In this study, we assessed the relative safety of wild *Cordyceps sinensis* (*C. sinensis*) by comparing its hepatorenal toxicity with that of a mixture of inorganic arsenic compounds (sodium arsenate and sodium arsenite) administered at an equivalent total arsenic dose. Despite containing inorganic arsenic, wild *C. sinensis* did not induce significant arsenic accumulation in major organs, including the heart, liver, spleen, lung, kidney, and brain, nor did it cause notable hepatorenal toxicity at its maximum clinical dose in long-term. In contrast, the inorganic arsenic compounds mixture led to marked arsenic accumulation in the liver and kidney, accompanied by evident organ dysfunction. These findings demonstrate that wild *C. sinensis,* when used at its clinically recommended dose, exhibits substantially lower toxicity and tissue arsenic burden compared to equivalent dose of inorganic arsenic compounds mixture, providing important evidence to support its relative safety.”

The original article has been updated.

