# Supplementary material for: Wild Cordyceps sinensis exhibits far lower arsenic accumulation and hepatorenal toxicity in mice compared to equivalent dose of inorganic arsenic
Source: Front Pharmacol. 2025 Jun 24;16:1625045. doi: 10.3389/fphar.2025.1625045 (PMC12235186; doi:10.3389/fphar.2025.1625045)
Supplement: Supplementary file 1 [file DataSheet1.docx]

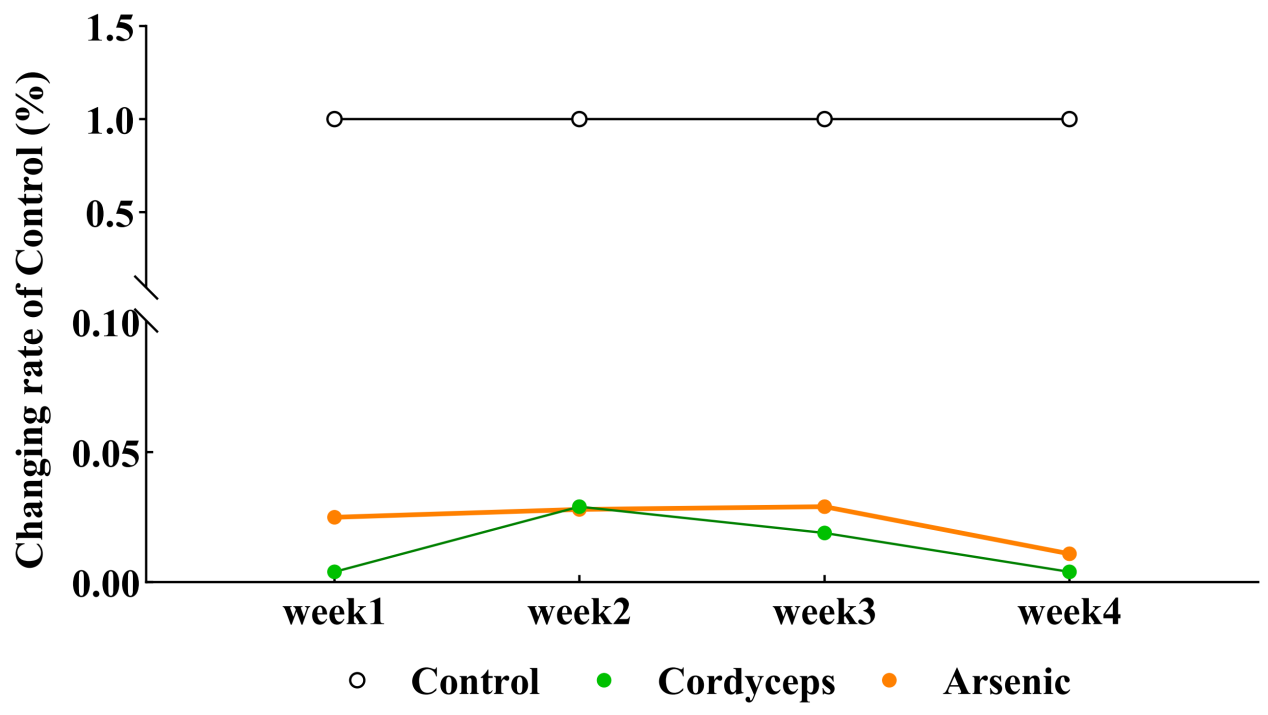


**Fig. S-1** Trends in body weight of mice over time after drug administration

Note: *n=6,* Y-axis: Relative body weight change of experimental groups vs. Control group at different time points*.*


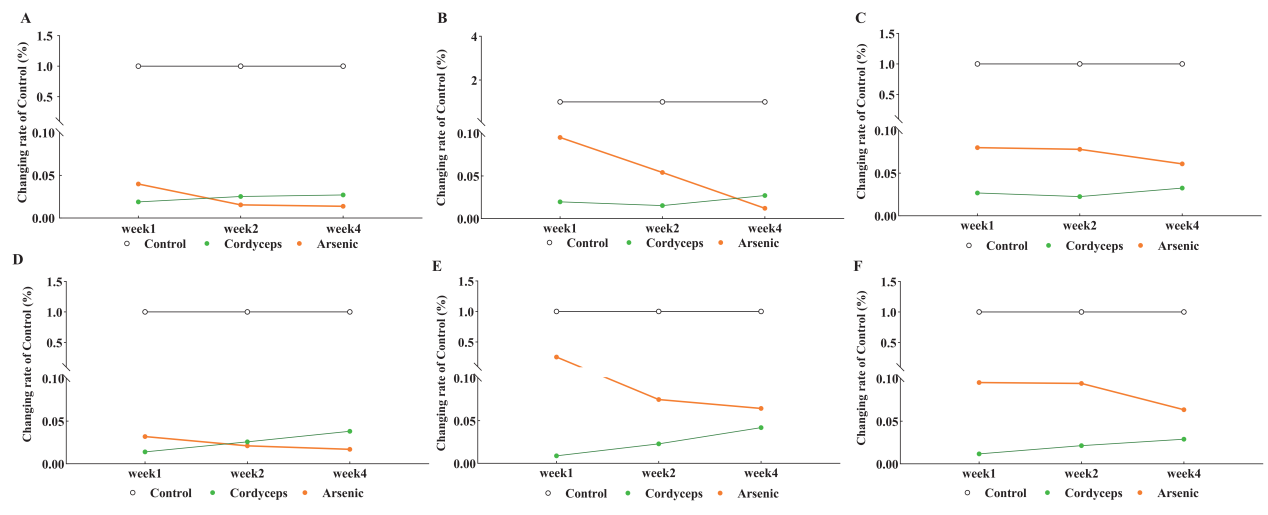


**Fig. S-2** Trends in organ-to-body weight ratio over time in mice after drug administration (A) Trends in Heart-to-body weight ratio after treatment; (B) Trends in Liver-to-body weight ratio after treatment; (C) Trends in Spleen-to-body weight ratio after treatment; (D) Trends in Lung-to-body weight ratio after treatment; (E) Trends in Kidney-to-body weight ratio after treatment; (F) Trends in Brain-to-body weight ratio after treatment.

Note: *n=6,* Y-axis: Relative changes in organ-to-body weight ratios of experimental groups vs. Control group at different time points.


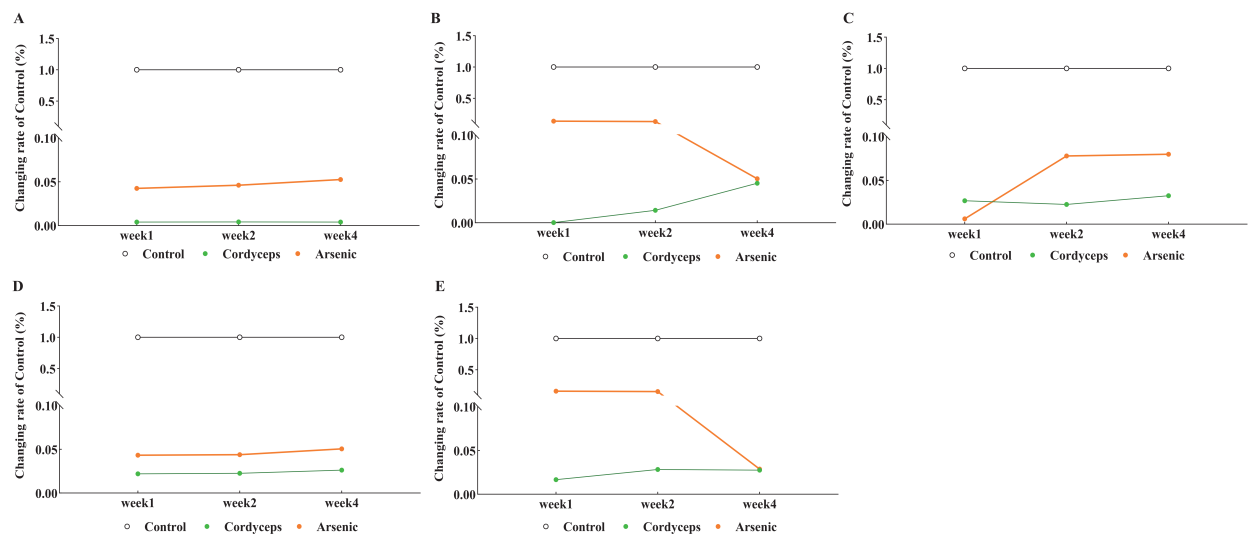


**Fig. S-3** Trends in organ-to-brain weight ratio over time in mice after drug administration (A) Trends in Heart-to-brain ratio after treatment; (B) Trends in Liver-to-brain ratio after treatment; (C) Trends in Spleen-to-brain ratio after treatment; (D) Trends in Lung-to-brain ratio after treatment; (E) Trends in Kidney-to-brain ratio after treatment.

Note: *n=6,* Y-axis: Relative changes in organ-to-brain weight ratios of experimental groups vs. Control group at different time points.


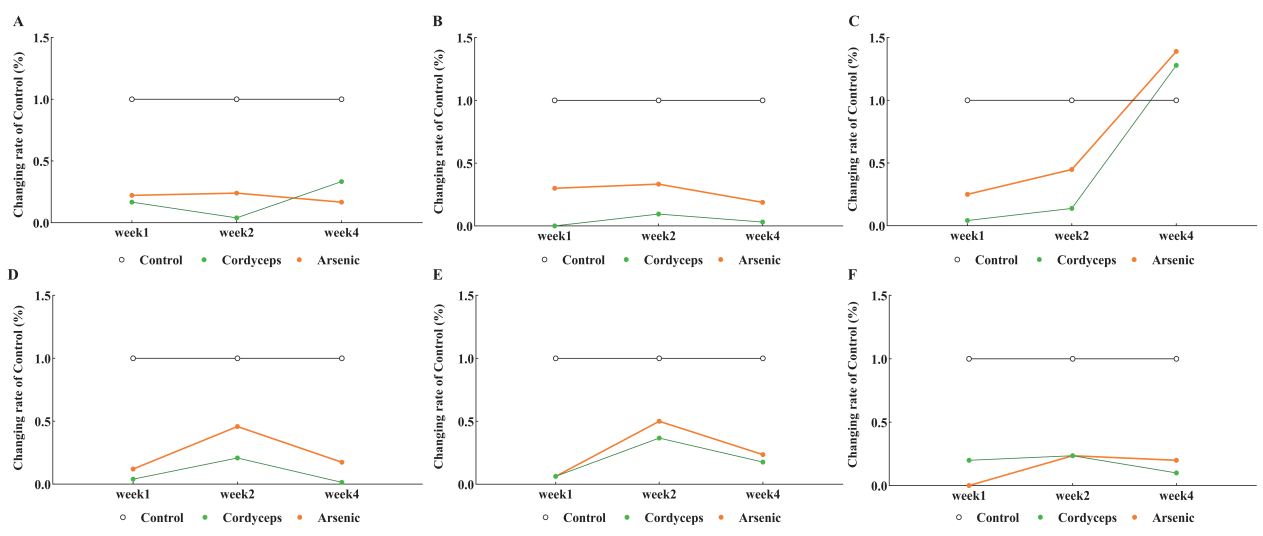


**Fig. S-4** Trends in organ arsenic levels over time in mice after drug administration

(A) Trends in arsenic content in heart after treatment; (B) Trends in arsenic content in liver after treatment; (C) Trends in arsenic content in spleen after treatment; (D) Trends in arsenic content in lung after treatment; (E) Trends in arsenic content in kidney after treatment; (F) Trends in arsenic content in brain after treatment.

Note: *n=6,* Y-axis: Relative changes in organ arsenic content of experimental groups vs. Control group at different time points.


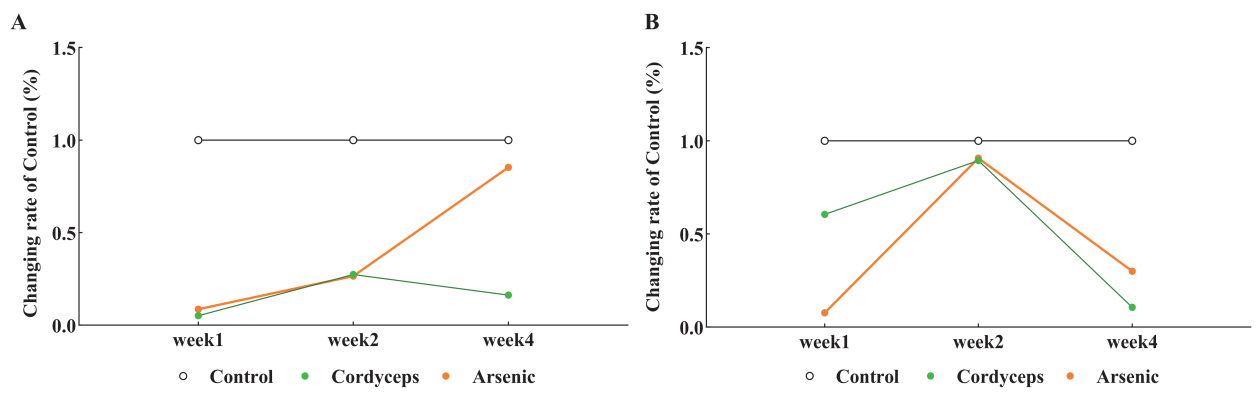


**Fig. S-5** Trends in AST and ALT over time in mice after drug administration (A) Trends in serum ALT levels in mice; (B) Trends in serum AST levels in mice

Note: *n=6,* Y-axis: Relative changes in serum AST and ALT levels of experimental groups vs. Control group at different time points.


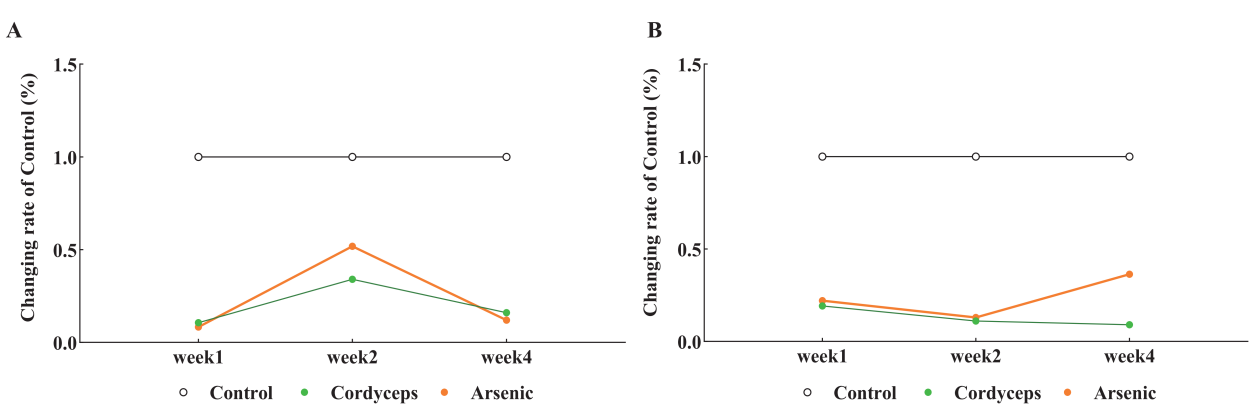


**Fig. S-6** Trends in BUN and CRE over time in mice after drug administration (A) Trends in serum CRE levels in mice; (B) Trends in serum BUN levels in mice

Note: *n=6,* Y-axis: Relative changes in serum BUN and CRE levels of experimental groups vs. Control group at different time points.
